# Trichotillomania as a Manifestation of Dementia

**DOI:** 10.1155/2016/9782702

**Published:** 2016-10-20

**Authors:** Pongsatorn Paholpak, Mario F. Mendez

**Affiliations:** ^1^Department of Neurology, David Geffen School of Medicine, University of California Los Angeles (UCLA), Los Angeles, CA 90095, USA; ^2^Department of Neurology, Neurobehavior Unit, VA Greater Los Angeles Healthcare System, Los Angeles, CA 90073, USA; ^3^Department of Psychiatry, Faculty of Medicine, Khon Kaen University, Khon Kaen 40002, Thailand

## Abstract

Pathological hair-pulling or trichotillomania, which is commonly associated with anxiety and depression, obsessive-compulsive disorder, and neurodevelopmental disorders, has been rarely associated with dementing illnesses. Investigators have not clarified the neural correlates and treatment of trichotillomania in dementia. We report a patient who developed an early-onset cognitive decline with genetic, cerebrospinal fluid biomarker and structural and functional neuroimaging studies consistent with Alzheimer's disease. Eight years into her disease, she developed severe, repetitive hair-pulling behavior leading to marked hair loss, along with other repetitive and “frontal” behaviors. Selective serotonin reuptake inhibitors (SSRIs) were ineffective in controlling her hair-pulling behavior, which subsequently responded to quetiapine 150 mg/day. This patient and a review of the literature suggest that trichotillomania may be a compulsive-related symptom in dementias of different etiologies as they involve frontal areas and release primitive grooming behavior from frontostriatal dysfunction. Dopamine blockade, rather than SSRIs, may be effective in managing trichotillomania in dementia.

## 1. Introduction

Trichotillomania is characterized by an overwhelming urge to pull out one's own hair with resultant noticeable hair loss. Although trichotillomania is a distinct diagnostic entity [1], in as many as 3 out or 4 patients it occurs along with a comorbid condition [2–4], most commonly depressive disorder followed by obsessive-compulsive disorder (OCD) [2–4]. Trichotillomania also occurs with other body focused repetitive behaviors (BFRB) such as excoriation or skin-picking and nail or cheek biting, in addition to repetitive hand-biting, head-banging, self-hitting, or lip-biting in neurodevelopmental disorders such as Lesch-Nyhan syndrome, Rett's syndrome, fragile X syndrome, autism, and mental retardation [1, 3].

Clinicians may be unware that trichotillomania can also be a neuropsychiatric symptom of dementia. Although the literature on this is sparse [5–7], trichotillomania can be a significant disability among patients with different dementing illnesses and a challenge for clinicians to effectively treat it. We describe a dementia patient with intractable trichotillomania, review what is known about this association, and discuss the possible etiology. This report is part of an Institutional Review Board approved study.

## 2.  Case Report

A 54-year-old, left-handed woman had a five-year history of a very insidious onset and gradually progressive decline in memory and cognition. Her husband reported early word-finding difficulty with increasing use of nonspecific words when she was not able to find the correct words. The patient also had impaired orientation, memory and new learning, auditory comprehension, and visuospatial abilities. The patient still retained the ability to participate in her activities of daily living, but she was unable to do them on her own. Her past medical history was negative for other diseases or toxic exposures, and there was no history of dementia in her family. On examination, the patient scored 9/30 on Mini-Mental State Examination (MMSE), and she had marked difficulty with language fluency, comprehension, and confrontational naming. Her declarative, episodic memory was significantly impaired. She could not copy visuospatial figures, search for dots and words, or perform executive tasks. Examination of cranial nerves, gait and coordination, motor, sensory, and reflexes did not reveal abnormalities.

**Figure 1:**
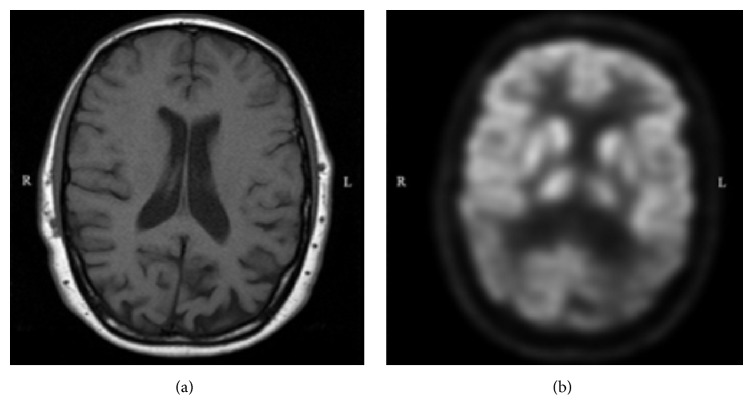
(a) T1 axial magnetic resonance imaging of brain shows predominant cerebral atrophy in the parietal lobes; (b) fluorodeoxyglucose positron emission tomography axial view showing bilateral parietal hypometabolism and hypometabolism of the posterior cingulate cortex.

The results of laboratory investigations were unremarkable except for the presence of an apolipoprotein (APOE) *ℇ*_4_ allele (*ℇ*_4_, *ℇ*_3_), a cerebrospinal fluid (CSF) biomarker profile consistent with Alzheimer's disease (AD), and corresponding changes on neuroimaging. CSF biomarkers included a *β*_42_-amyloid level of 379.6 pg/mL, a total tau level of 518.35 pg/mL, and a phosphorylated-tau level of 79.9 pg/mL. Magnetic resonance imaging (MRI) of the brain showed mild generalized atrophy, predominantly in the parietal regions, and positron emission topography (PET) scan showed significant hypometabolism in bilateral temporal and parietal lobes and the posterior cingulate cortex (see Figure 1). The patient was diagnosed with AD and treated with galantamine and memantine.

Eight years after the onset of her disease, she developed restless, agitated behavior, and repetitive, compulsive acts. The most prominent was severe, constant, and uncontrollable hair-pulling, which eventually resulted in her pulling out most of the hair on her scalp. Despite multiple attempts and trials of interventions and medications, her hair-pulling continued for the next two years. Her hair-pulling was constant during waking hours, unless restrained or distracted, and involved primarily her scalp, but also her eyebrows, arms, and other parts of her body. Once pulled she did not play with or eat the hair. Simultaneously, she developed rubbing and picking of her fingers to excoriation, hand-biting, and picking up lint from floor and shaping it into balls. The finger excoriation involved cutting at her thumb and other fingers with her fingernails until the affected fingertips bled. Her examination now included behaviors often found with frontal brain disease: palilalia or multiple repetitions of her own sparse utterances; logoclonia or repetition of end syllables; and upper extremity grasp reflexes often to the extent of “groping.”

Because of her dementia, she could not communicate a reason for her trichotillomania. Her husband reported that her insight regarding her illness was impaired, but she had obvious irritability and tension. The patient would become agitated when her husband attempted to stop her repetitive behaviors, but there was no evidence of relief after she successfully pulled out her hair. The trichotillomania and compulsive-like behaviors were not temporally associated with intercurrent illnesses or changes in medication. Trials of fluoxetine and sertraline (up to 150 mg/day for two or more weeks) were ineffective in decreasing her constant hair-pulling or her skin excoriation (per her husband's records of the length of waking time she spent on hair-pulling). The patient continued to worsen her repetitive behaviors; therefore, most of her medications were stopped and she was started on quetiapine at 25 mg/day, which was gradually titrated up to 150 mg/day. On this dose, both her hair-pulling and skin-picking decreased to intermittent episodes occurring during the week, and, most significantly, she was more easily diverted from these acts when they occurred. The patient has remained in this improved state for over a year of follow-up.

## 3. Discussion

This patient had profound trichotillomania in the context of an early-onset (<65 years of age) dementia with evidence for Alzheimer's disease (AD) on cerebrospinal biomarker studies and neuroimaging. The presence of an APOE*ℇ*_4_ allele may have facilitated her early age of onset of AD. Her trichotillomania occurred late in her disease, along with evidence suggestive of involvement or more frontal regions of the brain. Because of her advanced dementia, the patient was unable to give a reason for her hair-pulling behavior but did manifest restlessness, agitation, and other compulsive and self-injurious behaviors. In addition, there was no evidence of any delusional beliefs, medication changes, or intercurrent illnesses temporally related to her hair-pulling behavior. Trichotillomania has occurred in up to 3.6% of institutionalized elderly patients with moderate to severe dementia [8], but there have been few actual descriptions of trichotillomania as a manifestation of dementia. Most reported cases had an early-onset dementia, two with frontotemporal dementia [5, 6], one with vascular dementia [7], and our patient with AD.

Our understanding of the nature of trichotillomania is incomplete, and its occurrence in dementia may offer an avenue for understanding this behavior. Explanations for trichotillomania have ranged from an impulse control disorder such as kleptomania, a stereotypic self-injurious behavior as in neurodevelopmental disorders, a behavior that is part of the obsessive-compulsive disorder spectrum, or a purely body focused repetitive behavior (BFRB) along with skin-picking [9]. The concept of a release of innate grooming behavior may underlie all of these explanations for trichotillomania. BFRB are behaviors that remove parts of the body repetitively, such as pulling own hairs or picking on one's own skin, resulting in pleasurable stimulation, similar to the experience felt with normal grooming behavior [10]. Trichotillomania, skin-picking, and nail-biting and other BFRB may be extreme manifestations of normal grooming rituals which occur in many species and may be within the normal range of behavior [11]. Grooming behavior may be innate action patterns under frontostriatal control, associated with a heightened affective or pleasurable experience and evolved not only for hygiene and body caring, but also for helping in dearousal and stress reduction [12]. Both hair-pulling and skin-picking, which often occur together [3, 9, 13], may have impaired inhibitory control of grooming behavior from dysfunction of the right frontal gyrus, the anterior cingulate cortex (ACC), and white-matter tracts [14, 15]. Further support for this interpretation comes from genetic data with the* Sapap3* gene which codes for a protein that participates in the structure at glutamatergic synapses and is associated with disturbed frontostriatal circuits and excessive grooming behavior in humans as well as in mice [16].

The accompanying compulsive-like behaviors along with palilalia, logoclonia, grasp reflexes, and impairment of insight suggest frontal systems involvement with the progression of the dementia. When these compulsive-like behaviors occur near onset of the dementia, it may indicate behavioral variant frontotemporal dementia (bvFTD) and, when late, an extension of a dementia such as AD to frontal regions. Nearly 80 percent of bvFTD patients with proven frontal pathology have had compulsive-like behaviors, and neuroimaging and neuropathological studies indicate that compulsive-like behaviors in bvFTD patients result from damage to the frontal lobe and to the basal ganglia, particularly the caudate nuclei [17]. The literature also indicates that simple motor stereotypy (e.g., skin-picking, head rocking, and lip pursing) and complex motor stereotypy (e.g., hair-pulling, skin-picking, hand flapping, and wriggling with leg movement) involve frontostriatal disease or dysfunction [18].

Studies show that patients with trichotillomania have frontostriatal involvement and dysfunction. Compared to controls, patients with trichotillomania may have decreased volume of the left inferior frontal gyrus and left putamen volume [19, 20], suggesting dysfunction in a frontostriatal circuit. In contrast, other studies of trichotillomania show increased grey matter densities in frontal regions (e.g., right inferior and middle frontal gyri, ACC, and supplementary motor area), left striatum, or other related areas [21, 22]. Diffusion tensor imaging studies of white-matter tracts in patients with trichotillomania report decreased integrity in the ACC, presupplementary motor area, and temporal cortices and abnormalities in the frontostriatal-thalamic pathways [23, 24], particularly in association with longer duration and increased severity or hair-pulling [25]. Together, these and other neuroimaging studies implicate failure of cortical inhibition of motor responses or motor habits by frontostriatal-thalamic-cortical white-matter circuits in trichotillomania [26, 27]. In addition, functional MRI studies indicate that trichotillomania is associated with differences in brain activation depending on symptom provocation [28], with probable decreases in connectivity of part of this circuitry with reward and emotional areas of the brain [29]. All of these studies support a proposed mechanism for trichotillomania in dementia as resulting from damage to a frontostriatal network involved in motor response inhibition of innate urges to groom.

The neurochemical etiology of trichotillomania may involve different neurotransmitters. First, an imbalance of serotonin may play a role in repetitive self-injurious behaviors in both Cornelia de Lange syndrome, Lesch-Nyhan syndrome, and OCD. Some studies have shown that selective serotonin reuptake inhibitors (SSRIs), including fluoxetine, sertraline, escitalopram, and clomipramine, have successfully reduced the severity of compulsive-like behaviors in some patients with dementia [30] and have decreased the severity of trichotillomania along with reduced activity in frontal cortical and related regions [31]. One demented patient with trichotillomania responded well to citalopram 60 mg/day [6], whereas our patient, and another case [7], did not respond to multiple trials with SSRIs. Second, increased dopamine activity induced by dopamine agonist can increase repetitive self-injurious behavior in both animals and humans, while antipsychotics and other dopamine antagonists have been successful in reducing trichotillomania and other obsessive-compulsive behaviors [32, 33]. In our case, after we reduced sertraline, the patient seemed to have a good response to a moderate dose of quetiapine (150 mg/day). Paradoxically, many of atypical antipsychotics can also induce obsessive-compulsive behaviors through decreased serotonin activity [34]. Third, preclinical data and research in mice and in adults with intellectual disabilities show that naltrexone, an opioid antagonist, can help in reducing excessive grooming behaviors and self-injurious behaviors [12, 35]. Finally, there is significant evidence that N-acetylcysteine, which modulates glutamate, the main neurotransmitter in the fronto-striato-thalamic-cortical circuit, may have efficacy in treating trichotillomania [36–42].

In conclusion, trichotillomania can be a manifestation of compulsive-like, grooming behavior in dementias of different etiologies as they spread to involve frontal areas, with release of a frontal-striatal inhibitory pathway resulting in excessive primitive grooming behavior. Several medications can successfully treat trichotillomania. In dementia, if SSRIs are not effective, antipsychotic dopamine antagonists like quetiapine may reduce the incidence of trichotillomania [32], and N-acetylcysteine, which was not tried in this patient, may have therapeutic promise as well. This case report and review highlight the need to further elucidate the neurobiological basis of trichotillomania and its treatment in dementing illnesses.
